# The Application of 2,6-Bis(4-Methoxybenzoyl)-Diaminopyridine in Solvent Extraction and Polymer Membrane Separation for the Recovery of Au(III), Ag(I), Pd(II) and Pt(II) Ions from Aqueous Solutions

**DOI:** 10.3390/ijms22179123

**Published:** 2021-08-24

**Authors:** Daria Bożejewicz, Katarzyna Witt, Małgorzata A. Kaczorowska, Włodzimierz Urbaniak, Borys Ośmiałowski

**Affiliations:** 1 Faculty of Chemical Technology and Engineering, UTP University of Science and Technology, 3 Seminaryjna Street, PL 85326 Bydgoszcz, Poland; Katarzyna.Witt@utp.edu.pl (K.W.); Malgorzata.Kaczorowska@utp.edu.pl (M.A.K.); 2 Faculty of Chemistry, Adam Mickiewicz University in Poznań, 8 Uniwersytetu Poznańskiego Street, PL 61712 Poznań, Poland; Wlodzimierz.Urbaniak@amu.edu.pl; 3 Faculty of Chemistry, Nicolaus Copernicus University in Toruń, 7 Gagarin Street, PL 87100 Toruń, Poland; borys.osmialowski@umk.pl

**Keywords:** polymer membranes, solvent extraction, recovery of noble metal ions, 2,6-bis(4-methoxybenzoyl)-diaminopyridine, spectrophotometry

## Abstract

The work describes the results of the first application of 2,6-bis(4-methoxybenzoyl)-diaminopyridine (*L*) for the recovery of noble metal ions (Au(III), Ag(I), Pd(II), Pt(II)) from aqueous solutions using two different separation processes: dynamic (classic solvent extraction) and static (polymer membranes). The stability constants of the complexes formed by the L with noble metal ions were determined using the spectrophotometry method. The results of the performed experiments clearly show that 2,6-bis(4-methoxybenzoyl)-diaminopyridine is an excellent extractant, as the recovery was over 99% for all studied noble metal ions. The efficiency of 2,6-bis(4-methoxybenzoyl)-diaminopyridine as a carrier in polymer membranes after 24 h of sorption was lower; the percentage of metal ions removal from the solutions (%R_s_) decreased in following order: Ag(I) (94.89%) > Au(III) (63.46%) > Pt(II) (38.99%) > Pd(II) (23.82%). The results of the desorption processes carried out showed that the highest percentage of recovery was observed for gold and silver ions (over 96%) after 48 h. The results presented in this study indicate the potential practical applicability of 2,6-bis(4-methoxybenzoyl)-diaminopyridine in the solvent extraction and polymer membrane separation of noble metal ions from aqueous solutions (e.g., obtained as a result of WEEE leaching or industrial wastewater).

## 1. Introduction

Solvent extraction (SE), a process of separating metal ions from aqueous solutions by extraction into organic solvents immiscible with water, has been used for many years to recover various metals, both common and rare [1,2,3,4]. This well-established separation method has been widely used as a tool for the recovery of precious metals from different types of industrial waste, including waste electrical and electronic equipment (WEEE) [5,6,7]. This type of waste contains many valuable metals, and its recovery is often more effective than extraction from decreasing ore grades; thus, the term “urban mining” has been introduced [8]. The growing interest in methods of precious metals recovery from waste is related to the various applications of these materials. They can be used as raw materials for high-technology industries (e.g., electronic materials) as well as in pharmaceutics, jewelry, catalysis, etc. Furthermore, the demand for precious metals in industrial applications has significantly increased in the last few years [9,10]. 

Although extraction is a relatively simple method, the long time required for analysis and the need to use organic solvents (many of which are volatile and toxic) prove to be serious limitations. Moreover, some extractants are not effective or affordable enough [8]. It has been reported by Bulgariu and Bulgariu [11] that many of the disadvantages of classical SE can be minimized by using the aqueous two-phase system in the extraction process. They investigated the extraction of gold(III) in an aqueous PEG(1500)–(NH_4_)_2_SO_4_ two-phase system, using chloride ions as extracting agents. The PEG-based two-phase system they utilized was non-toxic, non-flammable and non-volatile. The obtained results showed that gold(III) was quantitatively extracted (>98%) into the PEG-rich phase in acid media. Another environmentally friendly two-phase extraction system based on polypropylene glycol 425 and sodium chloride has recently been successfully applied for the extraction of Pt(IV) and Pd(II) from diluted hydrochloric acid solutions [12]. Ecologically safe, two-phase aqueous systems have been used to extract various metal ions, not only noble ones. However, the utilization of such systems usually requires time-consuming determination of the appropriate process conditions (e.g., proper concentration of metal ions and other reagents used, temperature, pH) [13].

An alternative to the conventional method of liquid–liquid extraction is the application of membrane processes, e.g., polymer inclusion membranes (PIMs) [14], which can reduce the consumption of solvents. Other advantages of PIMs (a type of liquid membrane in which the liquid phase is held within the polymeric network, usually polyvinyl chloride (PVC) or cellulose triacetate) are their wide range of applications, high efficiency in the extraction of various metal ions, high stability and the possibility of conducting extraction and back-extraction processes [14,15]. It has been shown that the process of metal ion recovery using SE and PIMs depends on many factors, including the metal’s properties, concentration, pH, matrix complexity, etc., but the vital factor in both processes is the correct selection of the chemical compounds binding the metal ions (extractants and carriers, respectively) [15,16]. Hence, many studies conducted in recent years search for new extractants/carriers that would allow for more effective recovery of precious metals from aqueous and acidic solutions (e.g., obtained as a result of leaching of WEEE waste) [17,18]. A variety of chemical compounds, both commercially available carriers (e.g., Cyphos IL 101) and non-standard ones (e.g., niacin), have been used for this purpose [19,20,21,22,23,24]. Often, the application of the same compound as the extractant in SE and as the carrier in PIMs produces different results concerning the recovery of both heavy and noble metal ions [25,26].

**Figure 1:**
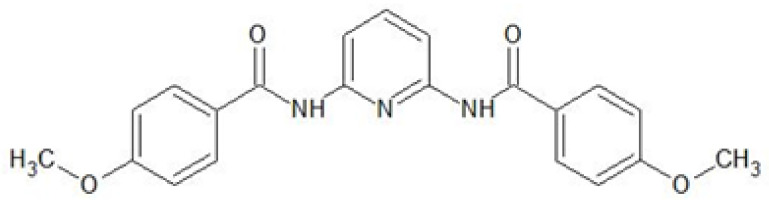
Structure of 2,6-bis(4-methoxybenzoyl)-diaminopyridine (C_21_H_19_N_3_O_4_).

This paper describes the results of the application of 2,6-bis(4-methoxybenzoyl)-diaminopyridine (shown in Figure 1) for the recovery of noble metal ions such as Au(III), Ag(I), Pd(II) and Pt(II) from aqueous solutions using SE and polymer membrane processes. 2,6-Bis(4-methoxybenzoyl)-diaminopyridine can be considered a heterocyclic amide which due to its structure possesses the ability to form complexes with various metal ions. In previous work, we showed that the presence of oxygen/nitrogen atoms in the molecule of 2,6-bis(4-methoxybenzoyl)-diaminopyridine allows it to form complexes with copper(II) ions; thus, it can be successfully used for the SE recovery of Cu^2+^ (recovery percentage above 99%) [27]. Despite the ability to form complexes with metal ions, so far 2,6-bis(4-methoxybenzoyl)-diaminopyridine has not been used as an extractant in liquid–liquid extraction designed for the recovery of noble metals ions, nor as a carrier in polymer membranes.

## 2. Results and Discussion

### 2.1. The Stability Constants of Complexes Formed by Precious Metal Ions with 2,6-Bis(4-Methoxybenzoyl)-Diaminopyridine

**Figure 2:**
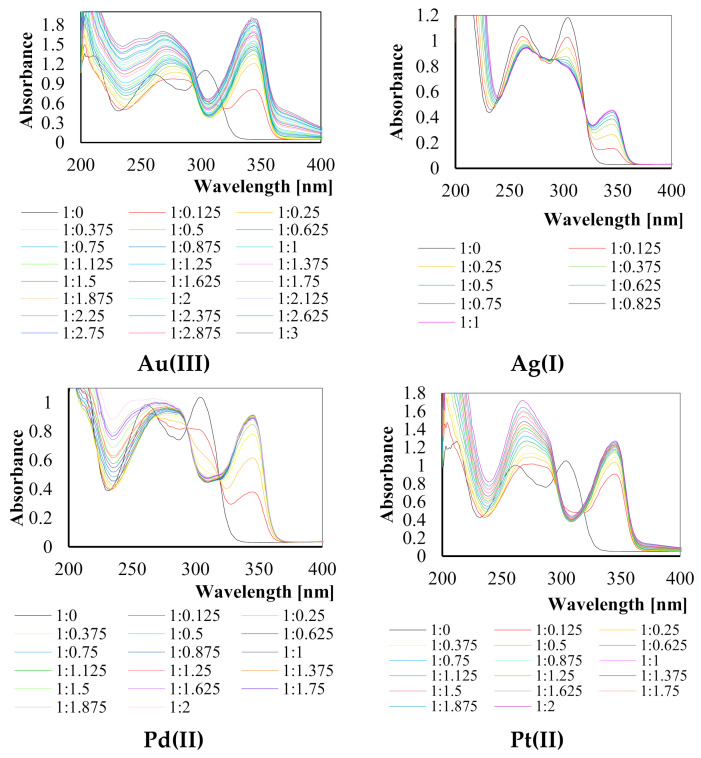
Absorption spectra of investigated systems of ligand to metal ions in various ratios (L:M).

For all investigated systems (ligand to chosen metal ion: Au(III), Ag(I), Pd(II) and Pt(II)) the absorption spectra were recorded in various ratios of L:M (Figure 2).

The obtained absorption spectra are characterized by absorption bands together with the isosbestic points in the UV region wavelengths ranging from 200 to 400 nm. Constant changes in the shapes of recorded spectra are related to the creation of new complexes with various ratios of ligand to the metal ion. Based on the above spectra, the stability constants of complexes of 2,6-bis(4-methoxybenzoyl)-diaminopyridine with Au(III), Ag(I), Pd(II) and Pt(II) ions were calculated and are presented in Table 1.

One valent, two valent and three valent metal ions created complexes of ligand to metal ion of type 1:1, 1:2 and 1:3, respectively. The most stable complexes were created by 2,6-bis(4-methoxybenzoyl)-diaminopyridine with platinum(II) ions. Only in the case of palladium(II) was its complex type 1:2 more stable than complex type 1:1, as the rest of the metal ions created exactly the most stable complexes (in which one metal ion is bound to one molecule of ligand). The values of the stability constants of complexes with two and three metal ions were lower. The values of the stability constants of complexes calculated previously with other metal ions were compared with the values obtained in this work and were equal for complex type 1:1 with Cu(II) ion 5.5 [27].

### 2.2. Classic Solvent Extraction

**Figure 3:**
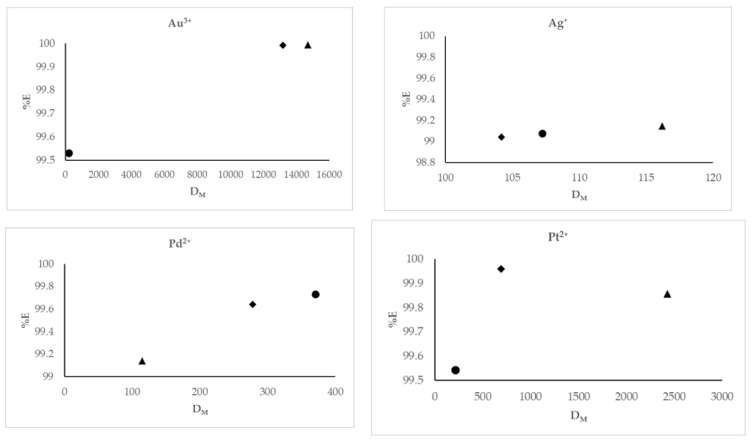
The dependence of the division ratio (D_M_) on the extraction percentage (%E), where the ● is related to the M:L ratio 1:1, the ▲ is related to the M:L ratio 1:5, and the ♦ corresponds to the M:L ratio 1:10. The given values of %E carry ± 0.03 and D_M_ carry ± 0.025.

The dependence of the parameters obtained after the solvent extraction process (i.e., division ratio on the extraction percentage) is shown in Figure 3.

If after the extraction process the sum of the concentration of metal ions in the organic phase is higher compared to the sum of the concentration of metal ions in the aqueous phase, the division ratio (D_M_) increases. The values of D_M_ impact the extraction percentage (%E), which is why the %E obtained for all extracted metal ions increased proportionally with increasing division ratios. The highest values of %E vs. D_M_ were for the ratio of M:L (metal:ligand) equaling 1:5 for gold, silver and platinum ions, while for palladium this value was the lowest.

**Figure 4:**
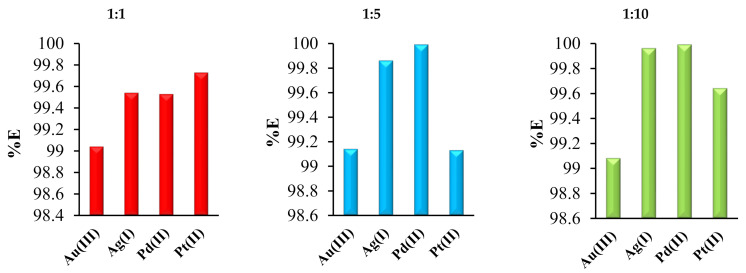
The extraction percentages obtained for various molar ratios of analyzed noble metal ions and 2,6-bis(4-methoxybenzoyl)-diaminopyridine (*L*, extractant).

Figure 4 shows the extraction percentages obtained for various molar ratios of analyzed noble metal ions and 2,6-bis(4-methoxybenzoyl)-diaminopyridine (*L*).

Application of 2,6-bis(4-methoxybenzoyl)-diaminopyridine in the SE processes led to the removal of approximately 99% of all noble metal ions from the aqueous solutions. The influence of molar ratios (M:L) on the amount of metal ions removed was less significant. The best (but not much better) results for metal ions recovery were obtained when the M:L ratio was 1:10. For example, the %E of palladium ions was 99.5%, 99.9% and 99.9% when the M:L molar ratios during the extraction were 1:1, 1:5 and 1:10, respectively. The differences were very small. Based on the results of these and previous studies [27], it can be concluded that 2,6-bis(4-methoxybenzoyl)-diaminopyridine is a very effective extractant that might be used for the recovery of various metal ions, including gold, silver, platinum, palladium and copper.

### 2.3. Membrane Extraction and Back-Extraction Processes

The sorption and desorption of metal ions on/from polymer membranes was conducted according to the method described by Witt et al. [28]. 

The sorption capacities q_t_ were calculated according to Equation (3) for gold(III), silver(I), palladium(II) and platinum(II) for 24 h sorption processes and are presented in Table 2.

The sorption capacity increased with the time of sorption. The highest q_t_ after 24 h of sorption process was observed for the membrane when the platinum ions were sorbed (q_t_ = 1.7516 mg/g), and the lowest was in the case of silver ions sorption (q_t_ = 0.1080 mg/g).

**Figure 5:**
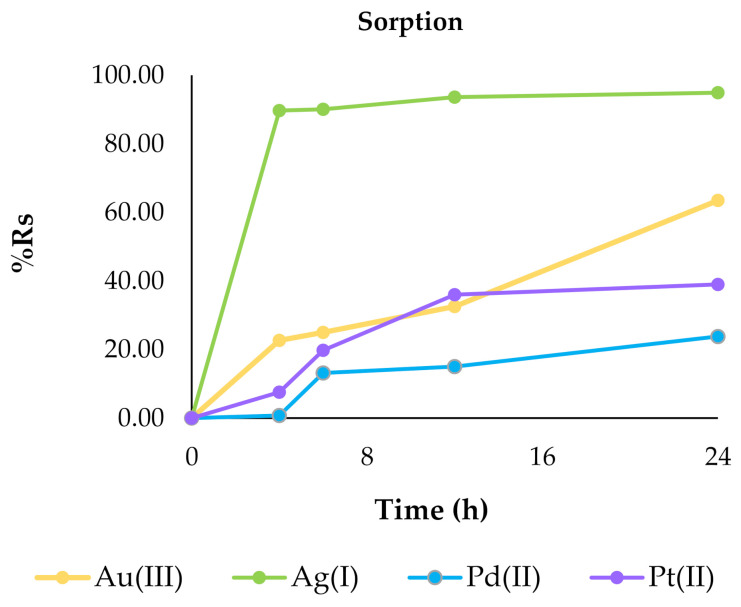
The percentage recovery of metals in the membrane (%R_s_) after 24 h with 2,6-bis(4-methoxybenzoyl)-diaminopyridine as a carrier for gold(III), silver(I), palladium(II) and platinum(II) ions. The given values of %Rs carry ± 0.019.

Figure 5 presents obtained results of %R_s_. 

The percentage of metal ion removal from the solutions (%R_s_) for all metal ions increased following time, and that dependency was similar to the changes of the sorption capacity q_t_ parameter. The %R_s_ parameter for the investigated metal ions after 24 h of sorption decreased in following order: Ag(I) (94.89%) > Au(III) (63.46%) > Pt(II) (38.99%) > Pd(II) (23.82%). In the case of sorption of silver(I) ions, equilibrium was reached after 6 h. For other metal ions a longer time was needed.

**Figure 6:**
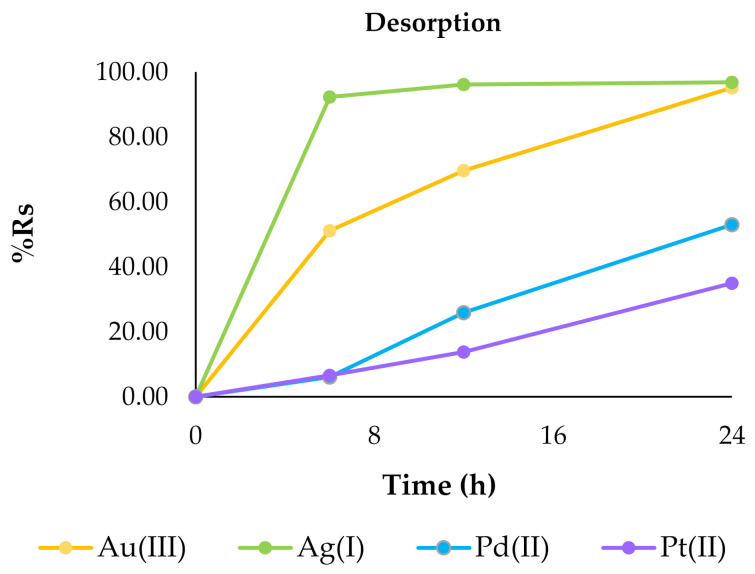
The sum of desorbed metal ions after 24 and 48 h. The given values of %R_des_ carry ± 0.019.

In addition to sorption, the opposite process was also carried out. For desorption of Au(III), Ag(I), Pd(II) and Pt(II) ions from the surface of polymer membranes containing 2,6-bis(4-methoxybenzoyl)-diaminopyridine a solution of 5 mol/dm^3^ of nitric acid was used. Figure 6 shows the percentage of metal ions desorbed as the sum of metal ions previously adsorbed on the surfaces of the membranes.

As a result of the desorption processes carried out, the metals adsorbed on the membrane surface were transferred into solutions. The metals were recovered into the concentrated solution, as its volume in relation to the solution used for sorption was three times smaller. The highest percentage recovery was observed after 48 h of desorption for silver (97.65%) and gold (96.62%) ions, whereas the lowest was for platinum (66.11%) and palladium (61.74%) ions. This confirms that by using membranes containing 2,6-bis(4-methoxybenzoyl)-diaminopyridine as a carrier it is possible not only to successfully adsorb metal, for example from wastewater, but also to transfer it to another concentrated aqueous phase and use this metal in further production processes.

**Figure 7:**
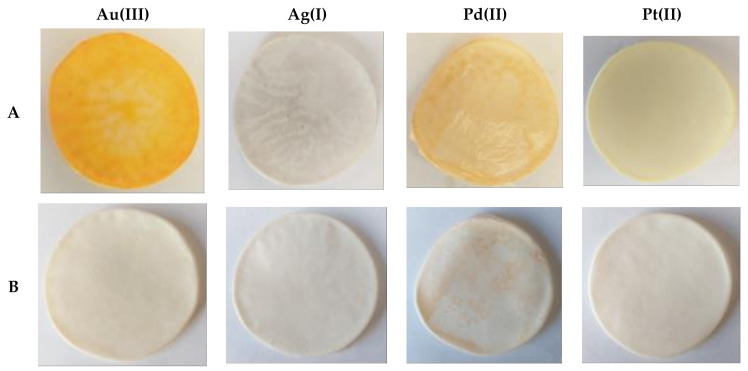
The polymer inclusion membranes containing 2,6-bis(4-methoxybenzoyl)-diaminopyridine after processes: membrane extraction (**A**), membrane back-extraction (**B**).

Figure 7 shows the polymer membranes after the membrane extraction processes and membrane back-extraction processes.

The above photos confirmed the efficiency of desorption processes, as it is clearly seen that metals adsorbed during sorption processes passed from the surfaces of the membranes (Figure 7A) into the solution of nitric acid. The surfaces of the membranes after desorption were colorless (Figure 7B).

## 3. Comparison of the Efficiency of Studied Processes

The results of the current research have shown that separation methods such as classic solvent extraction and membrane extraction are effective in recovering precious metals from aqueous solutions. The main aim of scientists involved in similar research is to find an effective extractant/carrier that will enable the high recovery of all precious metals. However, among the known extractants/carriers (Table 3), only 2,6-bis(4-methoxybenzoyl)-diaminopyridine enabled the effective recovery of gold, silver, palladium and platinum during the both the solvent extraction and membrane extraction processes.

## 4. Materials and Methods

### 4.1. Materials

2,6-bis(4-methoxybenzoyl)-diaminopyridine (*L*) (Figure 1) was synthesized following the procedure detailed in [27]. This compound is insoluble in water, but it is well soluble in organic solvents, e.g., chloroform, tetrahydrofuran, ethyl acetate or diethyl ether.

The other compounds used in the experiments, such as metal stock solutions with a concentration of 1000 mg/L (Au(III), Ag(I), Pd(II) and Pt(II)), concentrated nitric acid, ammonia, potassium hydroxide, chloroform and methanol were purchased from Avantor (Gliwice, Poland). All the reagents used in this work were of analytical grade and were used without further purification. Double-distilled water was used to dilute concentrated aqueous solutions. 

The pH of aqueous solutions was measured using a SevenCompact series pH meter (Mettler Toledo, Greifensee, Switzerland), which was calibrated using commercial technical buffer solutions (Mettler Toledo, Greifensee, Switzerland) having pH values of 2.00, 4.01, 7.00 and 10.00. The metal ion concentration in the aqueous phases was determined using the atomic absorption spectroscopy method (AAS 240FS Spectrometer, Agilent, Santa Clara, CA, USA).

### 4.2. Determination of Stability Constants of Complexes of Au(III), Ag(I), Pd(II) or Pt(II) with 2,6-Bis(4-Methoxybenzoyl)-Diaminopyridine

Stability constants (log K) of complexes of 2,6-bis(4-methoxybenzoyl)-diaminopyridine with Au(III), Ag(I), Pd(II) and Pt(II) ions were determined according to the previously described method [27]. For this purpose, a methanol solution of L with the concentration of 3 × 10^−5^ mol/dm^3^ and aqueous solutions of metal ions (M, each with a concentration of 100 mg/L) were prepared. When preparing solutions with different molar ratios of L:M, the appropriate higher amount of metal ion solution was added each time to a constant amount of ligand solution. The absorption spectra of obtained solutions were recorded, and stability constants of the created complexes of L with Au(III), Ag(I), Pd(II) and Pt(II) ions were calculated.

### 4.3. Separation Processes

**Figure 8:**
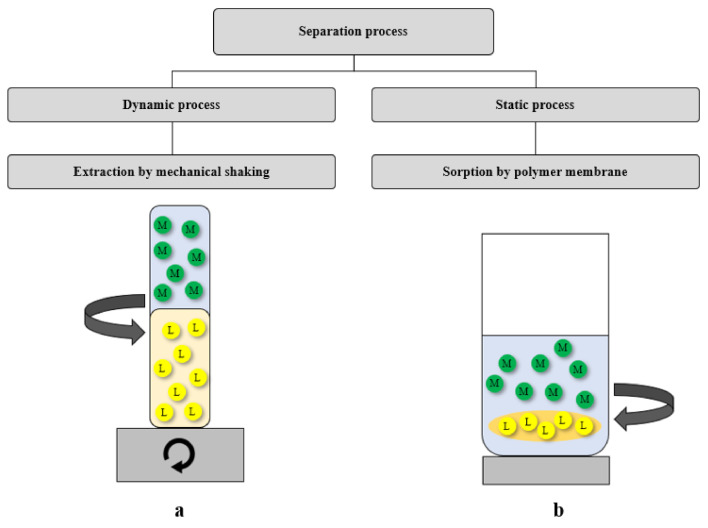
Scheme of the dynamic and static extraction processes, where *L*—2,6-bis(4-methoxybenzoyl)-diaminopyridine; (**a**) dissolved in the organic phase, (**b**) contained in a polymer membrane; M = Au(III), Ag(I), Pd(II) or Pt(II) ions in the aqueous phase.

The recovery of noble metal ions such as Au(III), Ag(I), Pd(II) and Pt(II) was carried out using 2,6-bis(4-methoxybenzoyl)-diaminopyridine in two different separation processes. The first process was classic solvent extraction (dynamic process), and the second was membrane extraction (static process) (Figure 8). Both processes resulted in separating metal ions from the aqueous phase.

For solvent extraction, 2,6-bis(4-methoxybenzoyl)-diaminopyridine (*L*) was dissolved in an organic solvent (chloroform), while in membrane extraction the ligand was immobilized in an organic polymer membrane.

#### 4.3.1. Classic Solvent Extraction

Classic solvent extraction experiments were performed at 25 ± 0.2 °C. The proper concentration of metal ions in the aqueous solutions was obtained by dilution of stock solutions of metal ions, the concentration of which was 1000 mg/L. The limit of concentration of metal ions in different samples was from 0.0004 to 0.0016 mol/dm^3^. The stock solution of the 2,6-bis(4-methoxybenzoyl)-diaminopyridine (*L*) had a concentration of 0.008 mol/dm^3^ in chloroform. The chloroform solution of *L* was added to the same volume of the aqueous solution. The volume of both phases (aqueous phase and organic phase) was 1100 µL. The molar ratios of the concentration of metal ions in aqueous solution to the ligand in organic solution were 1:1, 1:5 and 1:10, respectively. 

Prepared samples were then shaken for one hour. The equilibrium was established after approximately 30 min by visual inspection. Changes in the phase volumes were checked, the phases were then separated, and the pH and metal ion concentration of the aqueous phase were determined. Based on these measurements the parameters of solvent extraction, i.e., the extraction percentage (*%E*, Equation (1)) and the division ratio (*D_M_*, Equation (2)) were calculated.

The extraction percentage is a parameter that takes into account the partition coefficient and the volume of the aqueous (*V_aq_*) and organic (*V_org_*) phases. The division ratio is the ratio of the sum of the concentrations of all the substances in the organic phase (Σ[*M*]*_org_*) to the sum of the concentrations of all the substances in the aqueous phase (Σ[*M*]*_aq_*) [43].

(1) $$\%E=\frac{D_{M}}{D_{M+\frac{V_{aq}}{V_{org}}}}\times 100\%$$

(2) $$D_{M}=\frac{\Sigma {\left[M\right]}_{org}}{\Sigma {\left[M\right]}_{aq}}$$

#### 4.3.2. Membrane Extraction Processes

##### The Preparation of Polymer Membranes

**Figure 9:**
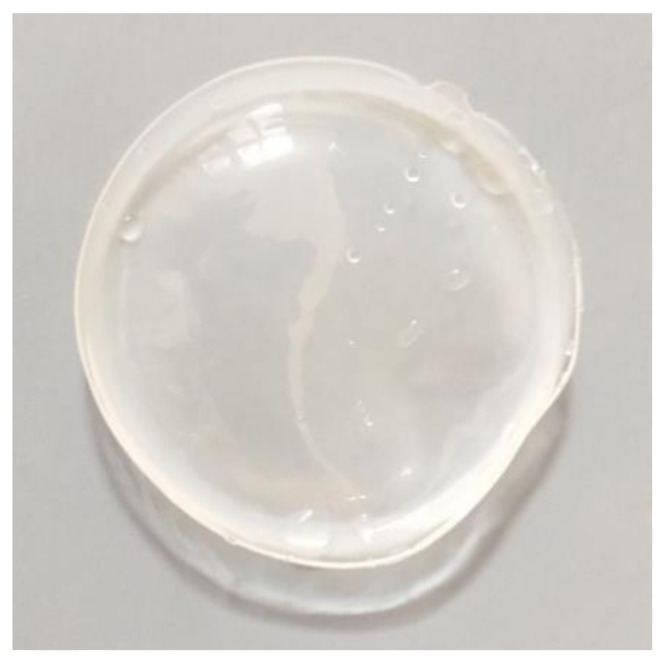
The polymer membrane with 20 wt.% of 2,6-bis(4-methoxybenzoyl)-diaminopyridine before the membrane extraction process.

To prepare the membranes, a solution containing 60 wt.% polyvinylchloride (PVC) as support, 20 wt.% a bis(2-ethylhexyl)adipate (ADO) as a plasticizer and 20 wt.% a 2,6-bis(4-methoxybenzoyl)-diaminopyridine as an ion carrier was prepared in 10 cm^3^ of tetrahydrofuran. Next, the solutions were poured onto ANUMBRA Petri dishes. After slow evaporation of the solvent for 24 h, the resulting polymer membranes (PMs) (Figure 9) were peeled off from the Petri dishes. Through the next 12 h, PMs were immersed in distilled water. The membranes were homogeneous, transparent, flexible and had good strength. The mean thickness of the membranes was determined as described previously [44]. The thickness of the membranes used for Au(III), Ag(I), Pd(II) and Pt(II) ion extraction was approximately 0.265 cm.

##### Membrane Extraction and Back Extraction Experiments

The circular polymer membranes were immersed in beakers containing one of the noble metal ions, such as Au(III), Ag(I), Pd(II) or Pt(II). The solutions of metal ions (volume 30 cm^3^) used as feed phase contained 3 mg/L of metal ions (pH solution was from 1.521 to 2.121). During the static processes, the metal ions from the solutions were bound to the surfaces of the membranes (sorption process). Small samples of well-defined volumes were taken from the solutions at regular intervals for 24 h to determine the concentration of metal ions in the solutions.

Analysis of the sorption processes of metal ions on membranes with 20 wt.% of 2,6-bis(4-methoxybenzoyl)-diaminopyridine as a carrier was performed using Equation (3):

(3) $$q_{t}=\left(\;\frac{c^{i}-c^{t}}{m}\right)\cdot V$$

where *q_t_*—the sorption capacity (mg/g), *V*—the volume of the solution (dm^3^), *m*—the mass of the membrane (g), and *c^i^* and *c^t^*—the analytical concentration of metal ions in the solution at the beginning and after a determined period of the sorption process (mol/dm^3^), respectively [45].

After 24 h of sorption, the percentage of metal ion removal from the solutions (*%R_s_*) was also determined (Equation (4)).

(4) $$\%R_{s}=\frac{c^{0}-c^{i}}{c^{0}}\times 100\%$$

where: *c*^0^ and *c^i^* denote analytical concentrations of metal ions in the solution at the beginning and after an appropriate period of the sorption process [28].

After extraction processes, the applied polymer membranes were immersed in 10 cm^3^ 5 mol/dm^3^ HNO_3_ solutions for 24 h to desorb metal ions from the membrane surfaces. The desorption efficiency (*%R_des_*) was calculated using Formula (5).

(5) $$\%R_{des}=\frac{c^{i}}{c^{a}}\times 100\%$$

where *c^a^* refers to the initially sorbed concentration of metals during the desorption process [28,46].

## 5. Conclusions

As a result of the performed separation processes, i.e., dynamic classic solvent extraction (mechanical shaking) and static membrane extraction (polymer membranes) using 2,6-bis(4-methoxybenzoyl)-diaminopyridine, it was found that this compound proved to be more efficient in SE processes. Presumably, shaking has a beneficial effect on the process of binding of noble metal ions by the studied compound (recovery of more than 99% of gold, silver, palladium and platinum ions). However, despite the high efficiency of this process, a relatively large amount of organic solvent (chloroform) was used, and the recovered metal ions remained bound to the extractant in the organic phase. Due to its sustainable chemistry, static membrane extraction appears to be the more advantageous process, despite the lower percentage recovery of metals from solutions. The recovery was the highest for silver (Ag(I) (94.89%)) and gold (Au(III) (63.46%)) ions, whereas for platinum and palladium ions it was much lower (Pt(II) (38.99%), Pd(II) (23.82%)). Moreover, the results of the performed desorption processes showed a high percentage of recovery, e.g., for gold and silver ions over 96% after 48 h. The reduction in the use of toxic solvents is important for economic and environmental reasons. The synthesis of 2,6-bis(4-methoxybenzoyl)-diaminopyridine is relatively cheap and simple. The obtained results show that 2,6-bis(4-methoxybenzoyl)-diaminopyridine may be potentially used in the future on a larger scale. One of the important areas of practical application of the tested compound may be the recovery of noble metal ions from solutions obtained as a result of WEEE leaching. The proposed methods (classic solvent extraction and polymer membrane extraction) may also be useful for the recovery of these metal ions from industrial wastewater.

## Figures and Tables

**Table 1:** Stability constants of complexes of 2,6-bis(4-methoxybenzoyl)-diaminopyridine with Au(III), Ag(I), Pd(II) and Pt(II) ions in various ratios of L:M.

Metal Ion	L—C_21_H_19_N_3_O_4_		
	(L:M = 1:1)	(L:M = 1:2)	(L:M = 1:3)
	log K_1_	log K_2_	log K_3_
Au(III)	5.540	4.851	4.607
Ag(I)	4.883	-	-
Pd(II)	4.917	5.398	-
Pt(II)	5.771	5.584	-

The given values of the log K carry ± 0.001 tolerance.

**Table 2:** The changes in sorption capacity of the membranes with 20 wt.% 2,6-bis(4-methoxybenzoyl)-diaminopyridine over time during sorption processes.

Metal Ions Time [h]	Au(III) q_t_ [mg/g]	Ag(I) q_t_ [mg/g]	Pd(II) q_t_ [mg/g]	Pt(II) q_t_ [mg/g]
4	0.3705	0.0326	0.0240	0.3794
6	0.3986	0.0388	0.3830	0.9588
12	0.5002	0.0923	0.4193	1.6790
24	0.9399	0.1080	0.6415	1.7516

The given values of the q_t_ carry ± 0.0015 tolerance.

**Table 3:** Selected metal ion extractants/carriers used in separation processes (SE, PM) for the recovery of noble metal ions.

**2,6-Bis(4-methoxybenzoyl)-diaminopyridine**						**D_2_EHAG**					
	Au(III)	Ag(I)	Pd(II)	Pt(II)	Ref.		Au(III)	Ag(I)	Pd(II)	Pt(II)	Ref.
SE	X	X	X	X	[This work]	SE	-	-	X	X	[29]
PM	X	X	X	X	[This work]	PM	X	-	-	-	[25]
**Kelex 100**						**Calix [4]pyrrole**					
	Au(III)	Ag(I)	Pd(II)	Pt(II)	Ref.		Au(III)	Ag(I)	Pd(II)	Pt(II)	Ref.
SE	-	-	X	X	[30,31]	SE	-	-	-	-	-
PM	X	-	-	-	[32]	PM	-	X	X	-	[23,33,34]
**Cyphos IL 101**						**Cyphos IL 102**					
	Au(III)	Ag(I)	Pd(II)	Pt(II)	Ref.		Au(III)	Ag(I)	Pd(II)	Pt(II)	Ref.
SE	X	-	X	-	[35,36,37]	SE	X	-	X	-	[35,36,37]
PM	-	-	X	-	[37]	PM	-	-	X	-	[37]
**Cyphos IL 104**						**Cyanex 302**					
	Au(III)	Ag(I)	Pd(II)	Pt(II)	Ref.		Au(III)	Ag(I)	Pd(II)	Pt(II)	Ref.
SE	-	-	X	-	[38]	SE	X	X	X	-	[39,40,41,42]
PM	X	-	X	-	[37,42]	PM	-	-	-	-	-

“X” indicates that the specified extractant/carrier can be used to recover the specified metal ions.

## Data Availability

Not applicable.
